# Data‐Driven Design of Mechanically Hard Soft Magnetic High‐Entropy Alloys

**DOI:** 10.1002/advs.202500867

**Published:** 2025-03-26

**Authors:** Mian Dai, Yixuan Zhang, Xiaoqing Li, Stephan Schönecker, Liuliu Han, Ruiwen Xie, Chen Shen, Hongbin Zhang

**Affiliations:** ^1^ Institute of Materials Science Technical University of Darmstadt Alarich‐Weiss‐Str. 2 Darmstadt Germany; ^2^ Department of Materials Science and Engineering KTH ‐ Royal, Institute of Technology Stockholm SE‐10044 Sweden; ^3^ Max Planck Institute for Sustainable Materials Max‐Planck‐Str. 1 Düsseldorf Germany

**Keywords:** density functional theory, high‐entropy alloys, high‐throughput calculations, machine learning, mechanically hard soft magnets

## Abstract

The design and optimization of mechanically hard soft magnetic materials, which combine high hardness with magnetically soft properties, represent a critical frontier in materials science for advanced technological applications. To address this challenge, a data‐driven framework is presented for exploring the vast compositional space of high‐entropy alloys (HEAs) and identifying candidates optimized for multifunctionality. The study employs a comprehensive dataset of 1 842 628 density functional theory calculations, comprising 45 886 quaternary and 414 771 quinary equimolar HEAs derived from 42 elements. Using ensemble learning, predictive models are integrated to capture the relationships between composition, crystal structure, mechanical, and magnetic properties. This framework offers a robust pathway for accelerating the discovery of next‐generation alloys with high hardness and magnetic softness, highlighting the transformative impact of data‐driven strategies in material design.

## Graphical abstract

The design of materials that are both hard and magnetically soft is crucial for advanced technologies. This study introduces a data‐driven approach to discover high‐entropy alloys with these properties. By analyzing over 1.8 million calculations, the framework identifies promising candidates and accelerates the discovery of next‐generation multifunctional materials.

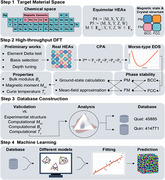

## 1 Introduction

Since the discovery of high‐entropy alloys (HEAs) in 2004,^[^ ^1^, ^2^ ^]^ these compositionally complex alloys have gained significant attention for their intriguing physical properties, largely attributed to four core effects.^[^ ^3^ ^]^ HEAs are renowned for their exceptional mechanical properties^[^ ^4^, ^5^, ^6^ ^]^ and also exhibit promising features, such as high magnetization, enabling their application as mechanically hard but magnetically soft magnets.^[^ ^7^ ^]^ Designing such alloys requires a thorough understanding of the interactions among mechanical strength,^[^ ^8^, ^9^ ^]^ thermal stability,^[^ ^10^, ^11^ ^]^ and intrinsic magnetic properties,^[^ ^8^, ^12^, ^13^ ^]^ in which elements such as Co, Fe, Ni, and Mn are indispensable to achieve sufficiently high saturation magnetization and low coercivity.^[^ ^14^, ^15^, ^16^ ^]^ Although some HEAs exhibit exceptional soft magnetic properties, many possess high hardness and a combination of features such as high saturation magnetization, electrical resistivity, and malleability. Conversely, traditional soft magnetic materials such as silicon steel and permalloy often exhibit high yield strength or hardness but suffer from brittleness.^[^ ^17^ ^]^ This contrast underscores the need for innovative design strategies to develop HEAs that integrate superior soft magnetic properties with enhanced mechanical strength.^[^ ^18^, ^19^, ^20^ ^]^

However, the vast compositional space of HEAs necessitates approaches beyond traditional experimental trial‐and‐error methods, which are both resource‐ and time‐intensive. In response, high‐throughput computational design (**HTP**)^[^ ^21^, ^22^, ^23^, ^24^ ^]^ has emerged as an effective solution that combines advanced thermodynamic and electronic structure methods. For example, HTP calculations using the CALPHAD method^[^ ^25^ ^]^ have been employed to predict phase diagrams for equimolar HEAs containing three to six elements, identifying 51 new alloy systems with potential for high‐temperature structural applications. The Lederer‐Toher‐Vecchio‐Curtarolo (LTVC) method^[^ ^26^ ^]^ integrates ab initio energy calculations with a statistical mechanical mean‐field model to estimate the transition temperatures, such as the Curie temperature and melting point, for solid‐solution HEAs. HTP calculations have also enabled the development of extensive databases. For instance, a recent study conducted 72 387 density functional theory (DFT) calculations on HEAs across 28 elements,^[^ ^27^ ^]^ mapping structural possibilities within the face‐centered cubic (FCC), body‐centered cubic (BCC), and hexagonal closed‐packed (HCP) phases. Additionally, the exact muffin‐tin orbital (EMTO) and coherent potential approximation (CPA) methods extend these high‐throughput techniques to calculate the elastic properties of 7086 cubic HEA structures within a set of 14 elements, including non‐equimolar compositions.^[^ ^28^ ^]^ Furthermore, a study investigating quaternary HEAs with solid‐solution phases of FCC and BCC covering 38 elements generated a large material database, incorporating properties such as magnetization, Curie temperature, and residual resistivity.^[^ ^29^ ^]^ While existing databases have advanced the field, they often cover only limited properties or compositions, leading to sampling imbalances in the compositional space. This highlights the need for a more extensive dataset that bridges mechanical and magnetic properties through HTP calculations.

To further accelerate the HEA design, integrating advanced machine learning (ML) algorithms with HTP data enables the efficient exploration of expansive compositional spaces.^[^ ^30^, ^31^, ^32^, ^33^ ^]^ For example, a recent study demonstrated the application of state‐of‐the‐art ML combined with feature engineering to predict phase formation in HEAs using a relatively small dataset,^[^ ^34^ ^]^ achieving superior accuracy compared to traditional methods. Beyond enhancing the capabilities of ML in materials science, the Deep Sets architecture has been employed to predict the elastic properties of HEAs.^[^ ^28^ ^]^ This model ensures permutation invariance, which is essential for accurately representing and analyzing element sets. Additionally, a novel ML framework combining random forest classifiers with moment tensor potentials has been developed to predict corrosion resistance,^[^ ^35^ ^]^ identifying HEA compositions with enhanced resistance properties. Another groundbreaking development is the HEA‐GAD‐TERM framework,^[^ ^36^ ^]^ which accelerates the discovery of new alloys by efficiently managing vast compositional spaces with sparse data. This framework led to the identification of two high‐entropy Invar alloys with exceptionally low thermal expansion coefficients, making them promising candidates for applications requiring minimal thermal expansion. While ML has had a transformative impact on HEA development, its effectiveness depends on the quality of training data. Sparse datasets limit model generalization and hinder the exploration of compositional spaces. To unlock the full potential of ML, it is crucial to construct well‐balanced datasets across a wide range of properties.

In this work, we conducted HTP calculations on magnetic quaternary and quinary HEAs derived from combinations of five base elements (i.e., Cr, Mn, Fe, Co, Ni) and 37 additional elements, focusing on equimolar compositions. For each combination, we evaluated the ground‐state energy and volume of both BCC and FCC structures, considering ferromagnetic and paramagnetic states. By assessing the bulk modulus as a mechanical property, alongside the total magnetization and Curie temperature as magnetic properties, we constructed a comprehensive database of multifunctional HEAs. As one of the most extensive datasets available, our database ensures uniform compositional sampling, addressing previous limitations. Integrating HTP data with ML enables a thorough exploration of the magnetic and mechanical properties of HEAs. Additionally, a user‐friendly website provides visualization, querying, and analysis tools, facilitating HEA research and collaboration.

## 2 Results

### 2.1 A Comprehensive Workflow for High‐Entropy Magnets

**Figure 1:**
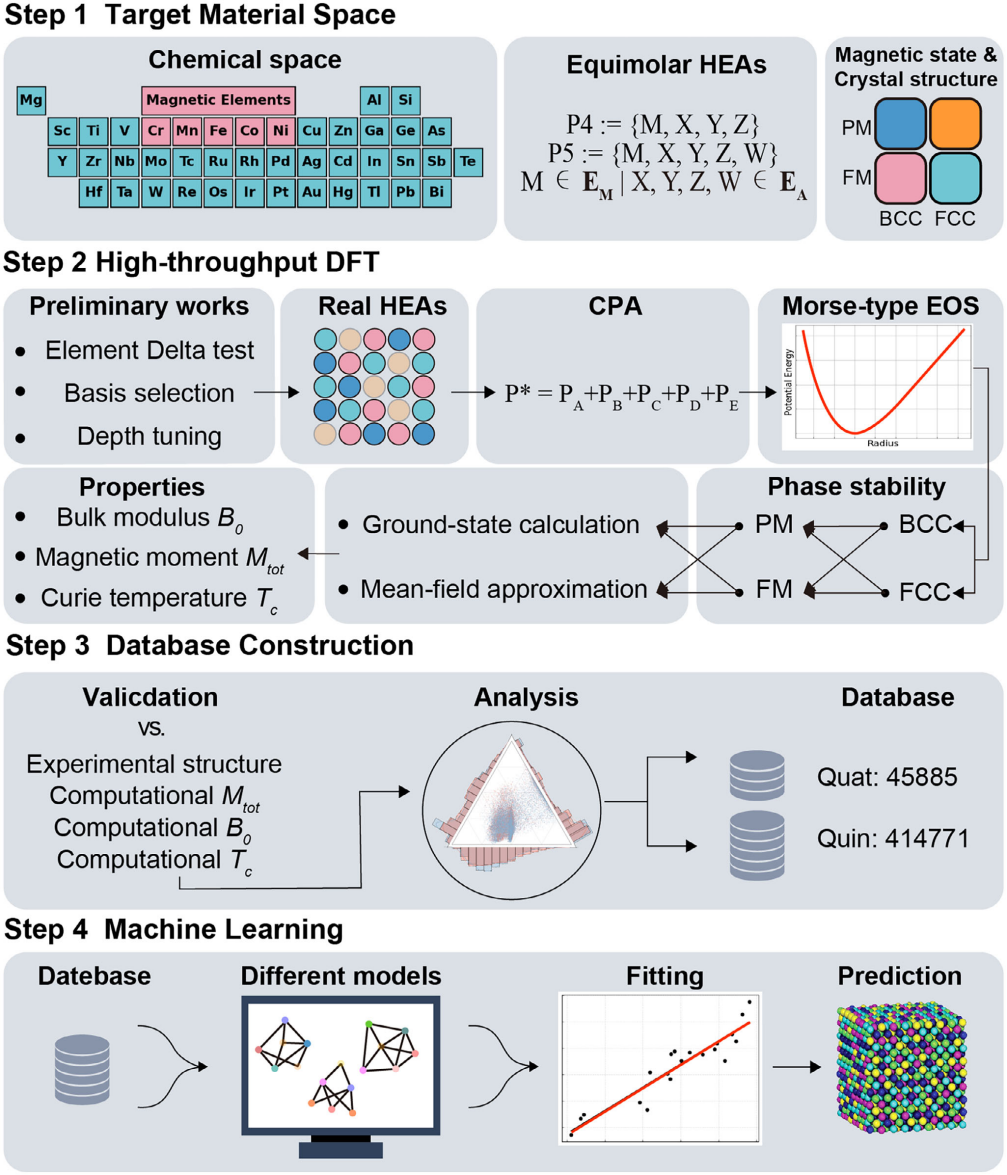
Workflow for HTP exploration and HEA property prediction. The schematic illustrates a comprehensive methodology for HEA discovery, beginning with the definition of the target material space and the strategic selection of magnetic and nonmagnetic elements. HTP‐DFT calculations follow, incorporating key steps such as the Δ‐test, basis set optimization, and modeling using the coherent potential approximation and Morse‐type equation of state. The results are validated, analyzed, and compiled into an extensive database, which is then integrated into machine learning models to predict HEA properties.

To explore the broad chemical space and uncover the fundamental principles governing HEAs, we conducted an exhaustive study of equimolar compositions consisting of both magnetic and nonmagnetic elements, as shown in **Figure** **1**. The chemical space includes 42 elements from the periodic table, collectively labeled as *E*_*A*_, with a focus on elements exhibiting long‐range magnetic order (Cr, Mn, Fe, Co, Ni), denoted as *E*_*M*_. HEA compositions are formed from equimolar proportions of four or five distinct elements, expressed as *P*_4_ = {*M*, *X*, *Y*, *Z*} and *P*_5_ = {*M*, *X*, *Y*, *Z*, *W*}, where *M* is selected from the set of magnetic elements *E*_*M*_ and {*X*, *Y*, *Z*, *W*} chosen from the 42‐element set *E*_*A*_. That is, all feasible configurations containing at least one magnetic element from *E*_*M*_ are considered, including {*M*, *M*, *X*, *Y*}, {*M*, *M*, *M*, *X*}, {*M*, *M*, *X*, *Y*, *Z*}, {*M*, *M*, *M*, *X*, *Y*}, and {*M*, *M*, *M*, *M*, *X*}. The computational analysis considers paramagnetic (PM) and ferromagnetic (FM) states, as well as BCC and FCC crystal structures.

Our HTP‐DFT workflow, implemented using the EMTO‐CPA method, enabled accurate calculations of structural and magnetic properties, including the Curie temperature and magnetic moment. Structural properties were determined using a Morse‐type equation of state (EOS), while Curie temperatures were estimated through a mean‐field approximation (MFA). More detailed information and a complete explanation can be found in Section 4. These results were thoroughly validated through comparison with both experimental and computational data, as outlined in the supplementary information (Tables S1–S5, Supporting Information). Based on this solid validation, we constructed an interactive database containing more than 460 000 HEA compositions, offering a valuable resource for exploring the vast design space. To enhance the predictive capabilities of the database, we employed ML ensemble models to predict phase stability and target properties. Coupled with dimensionality reduction techniques, this framework enabled us to extract the key compositional features driving HEA properties. The interactive database supports researchers in analyzing trends and identifying compositions that align with specific property criteria.

### 2.2 Interactive Database for High‐Entropy Magnets

**Figure 2:**
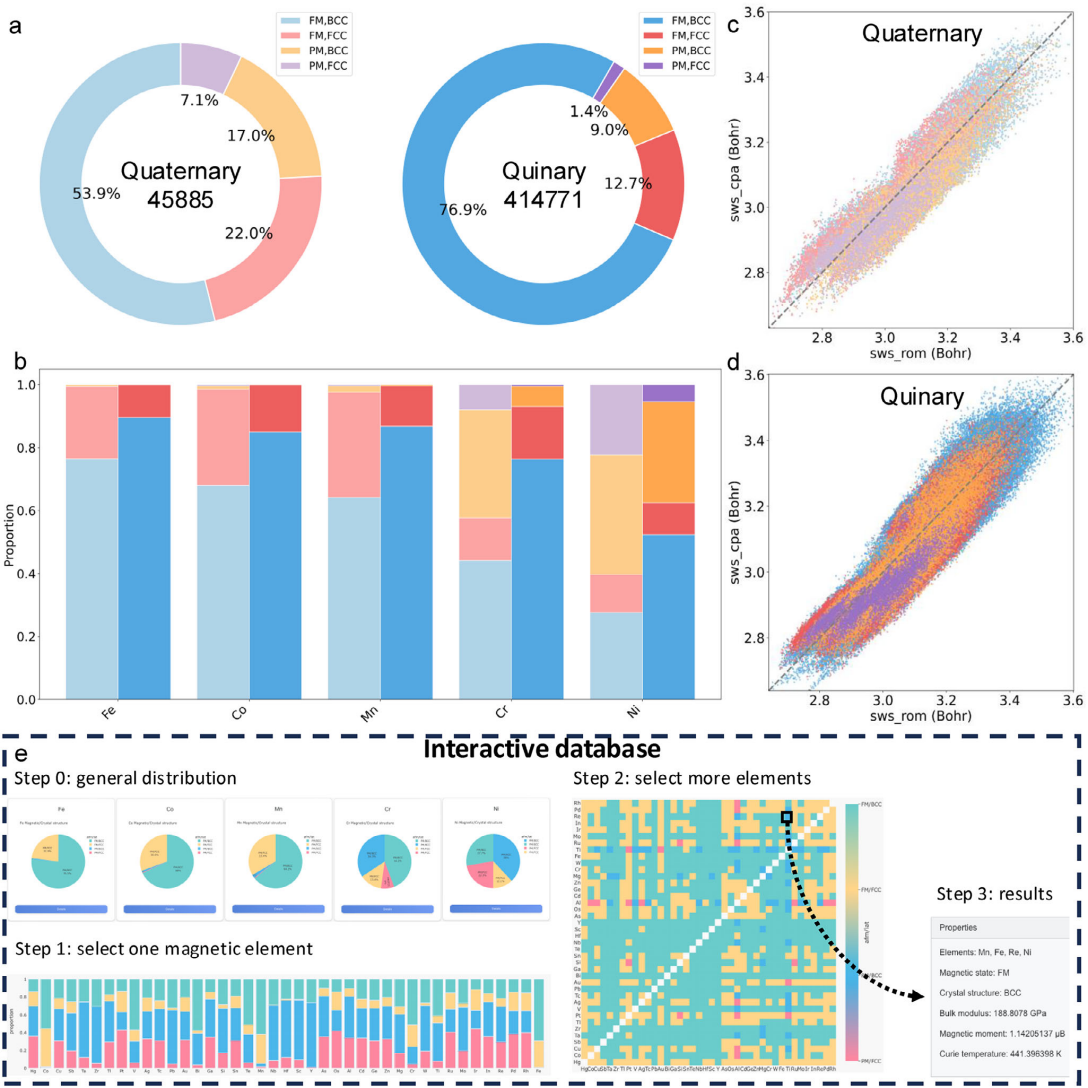
Overview of the HEA database and interactive analysis interface. Panel (a) presents the overall phase distribution of HEAs, classified by crystal structure and magnetic state, with quaternary and quinary sets distinguished by bright and dark colors, respectively. Panel (b) illustrates the relative prevalence of the four phases for the five primary magnetic elements using bar charts. Panels (c) and (d) compare the Wigner‐Seitz volumes predicted by the rule of mixtures with those obtained using the EMTO‐CPA method for quaternary and quinary HEAs. Panel (e) showcases the interactive analysis interface, which enables a stepwise exploration of the database.

The HEA database and its interactive analysis interface, presented in **Figure** **2**, provide a valuable tool for systematically exploring HEAs, encompassing 45 885 quaternary and 414 771 quinary compositions. Figure 2a offers an overview of the phase constitution in both quaternary and quinary HEAs, revealing distinct trends as the number of elements increases. Among the 45,885 quaternary compositions, the FM BCC phase is the most prevalent (53.9%), followed by FM FCC (22.0%), PM FCC (17.0%), and PM BCC (7.1%). In contrast, the quinary dataset of 414,771 compositions exhibits an even higher dominance of the FM BCC phase (76.9%), while FM FCC (12.7%), PM FCC (9.0%), and PM BCC (1.4%) phases account for smaller proportions. These results highlight the increasing dominance of FM and BCC phases as the number of elements increases.

The validation in Table S2 (Supporting Information) shows excellent general agreement in phase classification. However, a few exceptions were observed. In the quaternary set, FeReAgMo^[^ ^26^ ^]^ was misclassified due to a small energy difference of 0.002 Ry between the BCC and FCC phases. In the case of CoCrFeNiTi,^[^ ^37^ ^]^ the structure is misclassified as BCC, with a 0.0045 Ry energy difference. Similarly, CoCuFeNiPd^[^ ^37^ ^]^ is expected to have a BCC structure, but the energy difference between FCC and BCC is –0.0033 Ry. While these small energy differences provide an initial estimate of phase stability, a comprehensive assessment must also account for entropy contributions from vibrational, electronic, magnetic, and configurational effects at nonzero temperatures.^[^ ^38^ ^]^

Building on the overview of phase constitution, Figure 2b explores phase distributions for transition metals commonly incorporated in HEAs, including Fe, Co, Mn, Cr, and Ni. A closer examination reveals that Fe, Co, and Mn exhibit a pronounced dominance of the FM‐BCC phase, consistent with the overall trend observed in both quaternary and quinary datasets. In contrast, Cr and Ni display more complex phase behaviors. Cr shows significant contributions from both the PM‐FCC and PM‐BCC phases, indicating diverse phase stability. Notably, Ni exhibits a relatively balanced phase distribution between FM‐FCC and PM‐FCC phases, highlighting its distinct role in influencing the structural diversity of HEAs. These observations underscore the relationship between elemental selection and phase behavior, offering valuable insights for optimizing HEA compositions.

As demonstrated in Figure 2c,d, the Wigner–Seitz atomic volume (SWS) predicted by the rule of mixtures (ROM) is compared with values calculated using the EMTO‐CPA method for quaternary and quinary HEAs. A clear linear correlation is observed, indicating systematic agreement with Vegard's law. However, noticeable deviations highlight the inherent limitations of ROM in fully capturing atomic‐scale interactions. ROM tends to overestimate SWS due to its simplistic assumptions, which neglect critical chemical interactions and atomic relaxations. The discrepancy between EMTO‐CPA calculations and ROM predictions is ≈6%, underscoring the necessity of computing the ground‐state energy and volume to accurately determine the mechanical properties of HEAs. While ROM can offer a first‐level estimation of general trends, it falls short compared to ab initio calculations for precise property predictions. A specific example, presented in Table S2 (Supporting Information), is the CrFeCoNi alloy,^[^ ^39^ ^]^ which is consistently identified as FCC in both theoretical and experimental studies. The computed lattice constant for this alloy is 3.552 Å, closely aligning with the experimentally measured value of 3.568 Å.

To facilitate the exploration of this extensive dataset, Figure 2e presents an interactive interface designed to streamline the analysis of HEA properties. The workflow begins with the visualization of general phase distributions for individual elements using pie charts. In Step 1, users initiate their analysis by selecting a magnetic element, visualized through stacked bar charts that display corresponding phase distributions. In Step 2, users select an additional element via a heat map, enabling the identification of phase stability trends. Finally, Step 3 provides comprehensive property details, including magnetic state, crystal structure, bulk modulus, total magnetic moment, and Curie temperature. This interactive process enhances database accessibility, allowing researchers to gain novel insights and accelerate the design and discovery of HEAs.

### 2.3 Multifunctional HEAs

**Figure 3:**
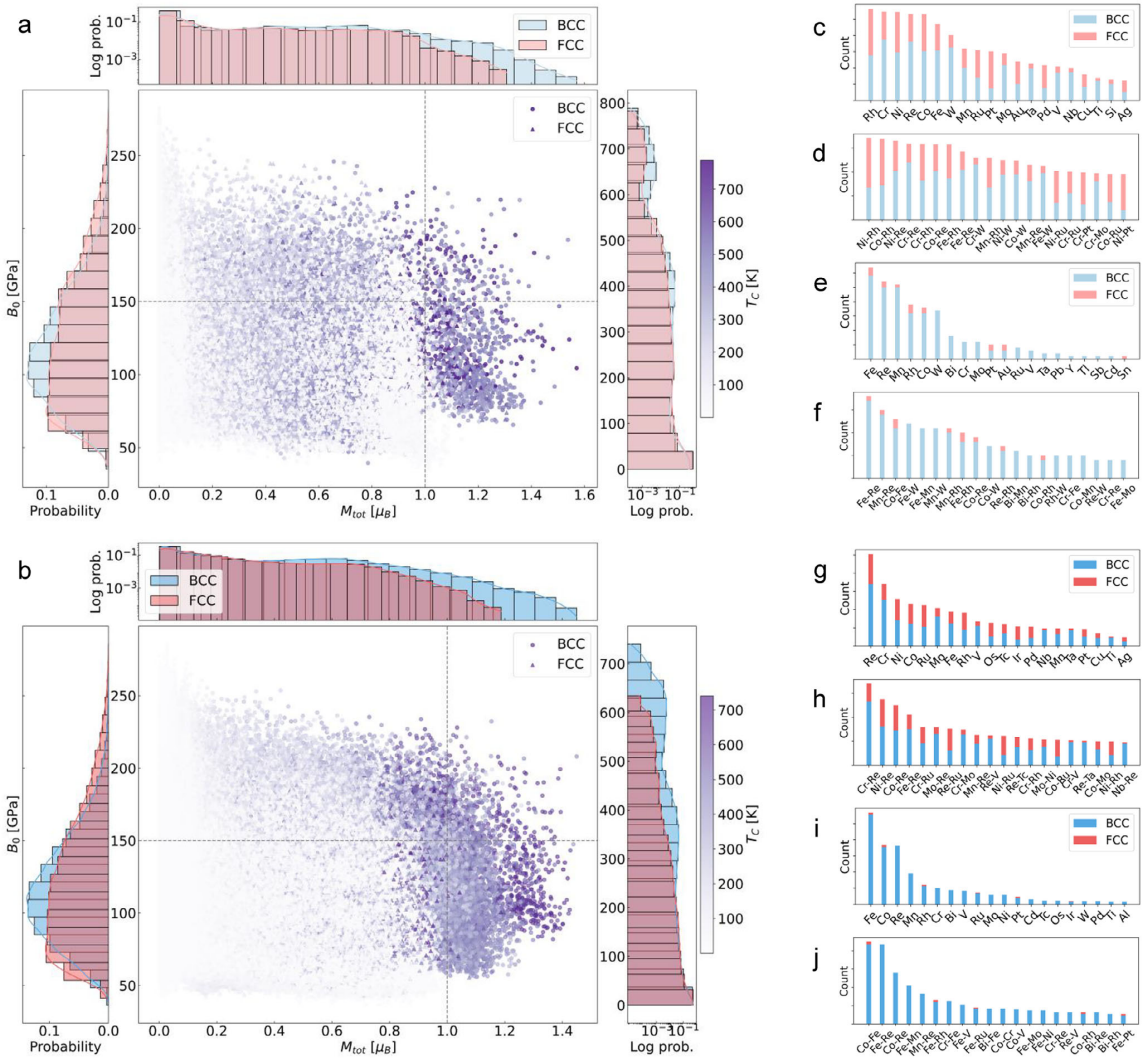
Distribution and correlation of target properties and elemental contributions in the HEA database. Panels (a) and (b) show the data points of *B*_0_, *M*_*tot*_, and *T*_*C*_ (color‐coded) for quaternary and quinary HEAs, with side panels showing the distribution of the properties for BCC (blue, circles) and FCC (red, triangles) structures. Panels (c–f) and (g–j) depict single and binary elemental contributions for quaternary and quinary HEAs, corresponding to the upper‐left (c,d,g,h) and upper‐right (e,f,i,j) regions of panels (a) and (b), respectively. Quaternary HEAs are represented in lighter shades, while quinary HEAs are shown in darker shades to differentiate their contributions.

**Figure** **3** presents the distribution and correlation between the magnetic and mechanical properties of HEAs with BCC and FCC crystal structures. The central regions of Figure 3a,b display scatter plots of bulk modulus (*B*_0_) versus total magnetic moment (*M*_*tot*_) for quaternary and quinary HEAs, respectively, with data points color‐coded by Curie temperature (*T*_*C*_). Most HEAs exhibit low *M*_*tot*_ and moderate *B*_0_ values. However, the inclusion of an additional element results in higher *M*_*tot*_ values and a noticeable increase in the spread of *B*_0_. This suggests that HEAs with more diverse elemental compositions tend to exhibit greater variability and potentially enhanced mechanical properties. For instance, CrNiMoW has a calculated bulk modulus of 225 GPa, closely aligning with the reported computational value of 245 GPa.^[^ ^28^ ^]^ The color bars reveal a broad variation in *T*_*C*_, particularly at higher *M*_*tot*_ values, reflecting diverse magnetic interactions within these HEAs. For example, FeCoCuNiPt is predicted to have a high Curie temperature of 858 K, closely matching the reported computational value of 837 K and the experimental value of 864 K.^[^ ^40^ ^]^

The left, upper, and right panels of Figure 3a,b illustrate the distributions of *B*_0_, *M*_*tot*_, and *T*_*C*_ in quaternary and quinary HEAs for BCC and FCC structures, respectively. The distribution plots reveal a clear grouping of properties based on crystal structure. BCC phases typically exhibit *B*_0_ values between 100 and 150 GPa, whereas FCC phases are predominantly distributed below 100 GPa, indicating that BCC structures generally possess higher *B*_0_ values on average. Regarding magnetic properties, the BCC phase consistently shows higher *M*_*tot*_ values in both quaternary and quinary HEAs. In the quaternary set, BCC structures demonstrate a slight advantage over FCC structures in the high *T*_*C*_ region, a trend that becomes more pronounced in the quinary set.

Figure 3c–f,g–j illustrate the contributions of individual elements and binary element pairs in quaternary and quinary HEAs, respectively. Specifically, panels (c) and (d) correspond to the upper‐left region of Figure 3a, while panels (e) and (f) represent the upper‐right region. Similarly, panels (g) and (h) map to the upper‐left region of Figure 3b, and panels (i) and (j) correspond to its upper‐right region. In both HEA sets, Re, Cr, Ni, Co, and Fe emerge as the predominant elements influencing material properties. As the system transitions from quaternary to quinary compositions, additional elements such as Mo, Ru, V, Os, Tc, and Ir are introduced, increasing compositional complexity in the targeted property regions. In the single‐element contribution maps, Rh exhibits a stronger influence in quaternary systems, whereas Re, Mo, and Ru become more prominent in quinary systems, suggesting a shift in elemental significance with increasing compositional diversity. Similarly, in the binary‐element contribution maps, Ni–Rh and Co–Rh pairs play a dominant role in quaternary HEAs, while Cr–Re, Mo–Re, and Mn–Re pairs become more frequent in quinary HEAs. These findings highlight how the introduction of additional elements alters the key compositional relationships that govern HEA properties, reflecting the increasing complexity and diversity of elemental interactions in multi‐component systems.

In regions characterized by high *B*_0_ but low *M*_*tot*_, compositions that favor FCC stabilization are more prevalent. This trend is particularly evident in alloys containing Ni, Co, Rh, and Ru. Among binary element interactions, Ni–Rh, Co–Rh, and Cr–Ru pairs frequently appear, indicating their significant role in promoting FCC stability. Conversely, in regions exhibiting both high *B*_0_ and high *M*_*tot*_, the dominant elemental contributions shift. Here, Fe, Mn, and W play a more influential role, while FCC‐stabilizing elements such as Rh become less significant. Instead, binary combinations such as Fe–Re, Mn–Re, and Cr–W appear more frequently, reinforcing BCC dominance in this compositional space. Additionally, interactions such as Cr–Mo, Mn–Re, and Mo–Re gain prominence in regions with high *M*_*tot*_, underscoring the increasing influence of refractory metals in stabilizing BCC structures. The screening results for the top 15 quaternary and quinary HEAs reveal distinct trends in mechanical and magnetic properties, as shown in **Tables** **1** and **2**. We recommend further investigation of these alloys to comprehensively characterize their properties.

**Table 1:** Screening top 15 results for equimolar quaternary HEAs with high bulk moduli, large total magnetic moment, and high Curie temperature.

System	Phase	*B*_0_ [GPa]	*M*_*tot*_ [μ_*B*_]	*T*_*c*_ [K]
MnFeReW	BCC	228	1.04	559
MnCrFeRe	BCC	220	1.20	523
MnFeReMo	BCC	220	1.28	570
MnFeRuW	BCC	213	1.30	513
TaFeCoRe	BCC	213	1.05	692
MnCrFeW	BCC	206	1.04	522
FeCoReNi	BCC	205	1.02	652
MnFeMoW	BCC	203	1.07	564
TaFeCoRh	BCC	201	1.02	679
FeCoRePb	BCC	199	1.22	648
MnFeCoRe	BCC	196	1.34	697
NbFeCoRh	BCC	195	1.01	701
TaMnFeRe	BCC	195	1.04	553
AlFeCoRe	BCC	193	1.01	674
FeCoPtAu	FCC	193	1.02	688

**Table 2:** Screening top 15 results for equimolar quinary HEAs with high bulk moduli, large total magnetic moment, and high Curie temperature.

System	Phase	*B*_0_ [GPa]	*M*_*tot*_ [μ_*B*_]	*T*_*c*_ [K]
CrFeCoReW	BCC	228	1.03	633
MnCrFeReW	BCC	223	1.02	552
CrFeCoReOs	BCC	222	1.03	555
FeCoReRhPb	BCC	215	1.05	603
CrFeCoReIr	BCC	211	1.06	566
V FeCoOsRh	BCC	209	1.01	634
MnFeCoReRu	BCC	208	1.18	623
FeCoReBiRh	BCC	207	1.07	545
FeCoSiReRu	BCC	205	1.01	606
AlFeCoReRu	BCC	205	1.06	632
MnCrFeCoRe	BCC	204	1.18	638
AlFeCoReRh	BCC	202	1.03	640
CrFeCoOsW	BCC	202	1.10	627
MnFeCoReRh	BCC	202	1.19	618
FeCoRePtPb	BCC	202	1.01	513

### 2.4 Machine Learning Modeling and Analysis of the Importance of the Characteristics of HEA

**Figure 4:**
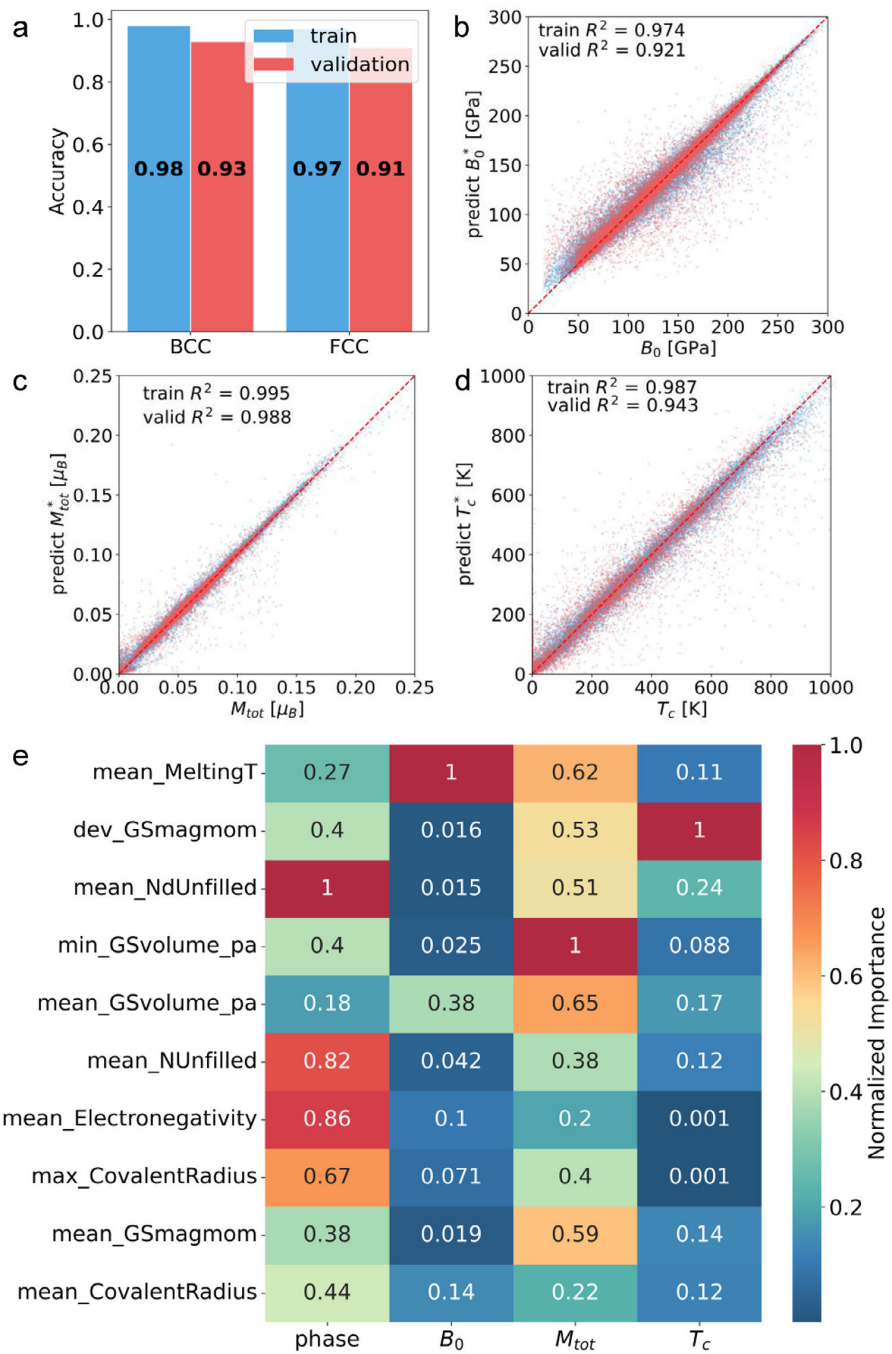
Machine learning performance and feature importance in predicting HEA properties. Panel (a) displays the predictive performance of the machine learning models for both BCC and FCC structures, demonstrating high *R* ^2^ values for both training and validation datasets. Panels (b–d) present scatter plots comparing the predicted and actual values of *B*_0_, *M*_*tot*_, and *T*_*C*_, respectively. The scatter plots reveal a strong correlation with minimal deviations from the red dashed line, which represents perfect prediction. Panel (e) illustrates the contributions of key input features to the prediction of target properties, including lattice structure (phase), *B*_0_, *M*_*tot*_, and *T*_*C*_. A detailed summary of these features is provided in Table S6 (Supporting Information).

**Figure** **4** presents the performance of our ensemble ML model, which has been trained on a set of key features for further importance analysis. The predictive performance of the model is evaluated using the precision of the lattice predictions and the *R* ^2^ value for three target properties. Figure 4a illustrates the accuracy of our model in distinguishing between BCC and FCC structures. In both the training and test datasets, the model achieved an impressive accuracy rate of more than 90%. Interestingly, performance in the test set is only 6% lower than in the training set, suggesting that the model generalizes effectively. Furthermore, the model exhibits a higher accuracy in predicting BCC structures than FCC ones. This difference can be explained by the fact that the data set for the FCC structures is only half the size of that for the BCC structures, as discussed in Section 4.

Figure 4b–d shows the model training and prediction results for *B*_0_, *M*_*tot*_, and *T*_*C*_, respectively. For the bulk modulus, the model demonstrated impressive *R* ^2^ values of 97.4% on the training set and 92.1% on the test set. For the total magnetic moment, the *R* ^2^ values on both the training and test sets exceeded 98%, suggesting that the model makes highly accurate predictions without overfitting. In terms of Curie temperature, the model achieved an *R* ^2^ value of 98.7% on the training data, and the performance of the test set was only 4.4% lower, indicating that the model generalizes well across different datasets. In general, our model shows strong predictive capabilities for both BCC and FCC structures and material properties.

Feature importance results, presented in Figure 4e, reveal the key descriptors governing the target properties of HEAs. The lattice structure is primarily influenced by electronic structure parameters, with mean_NdUnfilled (1.0) and mean electronegativity (0.86) playing dominant roles. *B*_0_ shows a strong correlation with melting temperature (1.0), underscoring its dependence on bonding strength. *M*_*tot*_ is mainly affected by atomic volume parameters (1.0, 0.65) and magnetic moment deviations (0.53, 0.59), highlighting the interplay between structural and electronic effects. *T*_*C*_ is predominantly governed by magnetic moment variation (1.0), indicating that spin interactions play a critical role in determining thermal stability.

## 3 Discussion

Our study integrates HTP‐DFT calculations with ML to establish a data‐driven framework for designing mechanically hard soft magnetic HEAs. By constructing a comprehensive database of 45 885 quaternary and 414 771 quinary HEAs, we overcome the limitations of previous studies constrained by small experimental datasets or low/medium‐throughput computations. This extensive dataset not only expands the compositional search space but also enables a more comprehensive and predictive assessment of phase stability, mechanical properties, and magnetic behavior. Crucially, our framework incorporates 42 elements, allowing us to capture complex multi‐element interactions with the DFT‐level accuracy. This capability reveals novel composition–property relationships that remain inaccessible through empirical models or traditional computational approaches, providing new insights into HEA design. Furthermore, our ML models demonstrate exceptional predictive performance, achieving *R* ^2^ > 0.9 for target properties. The identification of key atomic and electronic descriptors–such as electronegativity, covalent radius, and melting temperature–further deepens our understanding of the fundamental factors governing HEA properties. Together, these advances establish a robust and scalable framework for accelerating the discovery and optimization of high‐performance HEAs.

Our computational analysis reveals a systematic increase in the dominance of the FM‐BCC phase with greater elemental diversity, rising from 53.9% in quaternary to 76.9% in quinary HEAs. The BCC structure, with its lower atomic packing density and coordination number of 8, accommodates atomic size differences (δ) more effectively than the FCC structure, which has a coordination number of 12.^[^ ^41^ ^]^ Additionally, the valence electron concentration (VEC) of the BCC phase is more conducive to FM ordering, as evidenced in the CrMnFeCoNi system.^[^ ^42^ ^]^ As the number of elements increases, VEC values shift toward a range that stabilizes the FM‐BCC phase. Furthermore, the broader atomic size distribution enhances the stability of BCC phases, consistent with observations of Al‐induced FCC‐to‐BCC transformations.^[^ ^43^ ^]^

To optimize both mechanical and magnetic properties, we identify Co, Fe, Ni, and Mn as key contributors to achieving a high total magnetic moment, while Re and W significantly enhance mechanical strength through strong bonding interactions. Additionally, Mn and Fe further improve magnetic performance via strong exchange interactions, whereas Cr and Ni play a critical role in phase stability, influencing both mechanical and magnetic behavior. Structurally, our results confirm that BCC‐phase HEAs enriched with Re, W, and Fe exhibit higher intrinsic strength due to their ability to accommodate large atomic size mismatches and promote lattice distortion effects, making them ideal for high‐performance structural applications. Beyond composition–property relationships, increasing compositional complexity from quaternary to quinary HEAs enables finer tuning of mechanical and magnetic properties, thereby expanding design flexibility for targeted applications.

HEA toughness is fundamentally governed by microstructural features,^[^ ^44^ ^]^ including grain boundaries, phase distribution, and defect interactions. Among various intrinsic strength indicators, the bulk modulus plays a pivotal role in determining toughness,^[^ ^45^, ^46^ ^]^ as it reflects the alloy's inherent resistance to deformation. Nevertheless, our high‐quality, scalable dataset extends beyond soft magnetic HEAs, providing a robust, data‐driven foundation applicable to the design of high‐temperature and corrosion‐resistant alloys. By complementing experimental approaches, this dataset can further accelerate ML‐driven property predictions, enabling more efficient HEA design across a range of applications.

## 4 Computational Methods

### 4.1 High‐Throughput DFT

The computational workflow used HTP‐DFT to systematically evaluate the properties of equimolar HEAs. The DFT calculations were performed using the generalized gradient approximation for the exchange‐correlation functional, specifically applying the Perdew–Burke–Ernzerhof formulation.^[^ ^47^ ^]^ The Kohn–Sham equations were solved using the EMTO method,^[^ ^48^, ^49^ ^]^ with the chemical disorder treated via the CPA.^[^ ^50^ ^]^ A scalar‐relativistic approximation combined with a soft‐core basis scheme was applied, and total energies were calculated using the full charge density technique^[^ ^51^ ^]^ within an optimized overlapping muffin‐tin potential framework to enhance computational accuracy.

The EMTO method incorporated atomic‐like orbitals (s, p, d, and f states) to effectively capture electronic structure contributions from elements with significant d‐ and f‐orbital interactions. Brillouin zone sampling employed an equidistant 17 × 17 × 17 k‐point grid, optimized through convergence testing to ensure precision in energy and electronic density calculations. Calculations for the BCC and FCC lattices were performed on a minimum of eleven volumes, with energy‐volume data fitted to a Morse‐type EOS^[^ ^52^ ^]^ to determine the bulk modulus and phase stability. The robustness of the EMTO method for single‐element systems was validated using the Δ‐test, as detailed in Table 1 (Supporting Information), confirming its accuracy in predicting elemental ground‐state properties across structural variations.

Real HEA systems were modeled using the CPA to address compositional disorder, representing the alloy as an effective medium, P*, defined as a self‐consistent weighted combination of the constituent elements *P*_*A*_, *P*_*B*_, *P*_*C*_, *P*_*D*_, and *P*_*E*_. Magnetic properties were assessed by considering both FM and PM states. The PM state was simulated using the disordered local moment approximation,^[^ ^53^ ^]^ with *T*_*c*_ estimated using Equation 1. The MFA approach, based on ab initio energy differences and simplified models from previous studies,^[^ ^54^, ^55^ ^]^ balances computational efficiency and accuracy.

(1) $$k_{B}T_{C}^{MFA}=\frac{2}{3(1-c)}\left(E_{DLM}-E_{FM}\right)=\frac{2}{3(1-c)}\Delta E$$

for *E*_*DLM*_ − *E*_*FM*_ > 0, and

(2) $$T_{C}^{MFA}=0$$

otherwise, where *k*_*B*_ is the Boltzmann constant, *c* is the fraction of non‐spin‐polarized species, and *E*_*DLM*_ and *E*_*FM*_ represent the total energies of the DLM and FM states, respectively.

### 4.2 Database Construction

The database construction began with a validation process, where *B*_0_, *M*_*tot*_, and *T*_*C*_ were compared against available experimental and computational data to ensure accuracy and reliability (see Tables S2–S4, Supporting Information, for details). Following validation, data analysis was conducted using an interactive platform to identify trends and correlations across the compositional space. The validated data were then organized into a structured database comprising 45 885 quaternary and 414 771 quinary HEA compositions. This database serves as a vital resource for predictive modeling, facilitating the accelerated discovery and design of novel HEAs.

### 4.3 Machine Learning

In this study, we utilized an HTP‐DFT database to develop predictive models for HEA properties. The AutoGluon‐Tabular framework,^[^ ^56^ ^]^ known for its effectiveness in handling heterogeneous data and ensemble learning, was employed to enhance predictive accuracy. To effectively capture the complexity of HEA data, we applied advanced ensemble algorithms,^[^ ^57^, ^58^ ^]^ integrating predictions from K‐nearest neighbors, boosted decision trees, random forests, and neural networks. The dataset was randomly split into a training set (80%) and a test set (20%) to ensure robust model performance evaluation.

The feature selection strategy was guided by HTP computational methodologies^[^ ^59^ ^]^ and followed a general‐purpose framework for compositional descriptors.^[^ ^60^ ^]^ The selected features include a wide range of atomic, structural, electronic, and thermodynamic properties derived from both first‐principles calculations and experimental data. These include atomic radius, electronegativity, and valence electron count, as detailed in Table S6 (Supporting Information). Additionally, statistical descriptors, such as minimum, maximum, and standard deviation, are incorporated to capture the distribution of these properties across different elements. Dimensionality reduction was performed using principal component analysis, retaining the top 10 features that contribute most significantly to data variance. To further assess feature importance, gradient boosting trees were employed,^[^ ^61^ ^]^ enabling the identification of key descriptors with the greatest impact on predictive accuracy beyond variance‐based selection criteria.

## Conflict of Interest

The authors declare no conflict of interest.

## Supporting information

Supporting Information

## Data Availability

The data that support the findings of this study is available in the supplementary material of this article.
