# Supplementary material for: Data‐Driven Design of Mechanically Hard Soft Magnetic High‐Entropy Alloys
Source: Adv Sci (Weinh). 2025 Mar 26;12(19):2500867. doi: 10.1002/advs.202500867 (PMC12097130; doi:10.1002/advs.202500867)
Supplement: Supplementary file 1 — Supporting Information [file ADVS-12-2500867-s001.pdf]

ADVANCED SCIENCE
Open Access

Supporting Information

for *Adv. Sci.*, DOI 10.1002/advs.202500867

Data-Driven Design of Mechanically Hard Soft Magnetic High-Entropy Alloys

Mian Dai, Yixuan Zhang, Xiaoqing Li, Stephan Schönecker, Liuliu Han, Ruiwen Xie, Chen Shen* and Hongbin Zhang*

Supplementary Information for "Data-driven design of mechanically hard soft magnetic high-entropy alloys"

Mian Dai Yixuan Zhang Xiaoqing Li Stephan Schönecker Liuliu Han Ruiwen Xie Chen Shen* Hongbin Zhang*

Mian Dai, Yixuan Zhang, Ruiwen Xie, Chen Shen, Hongbin Zhang
Institute of Materials Science, Technical University of Darmstadt, Alarich-Weiss-Str. 2, Darmstadt, Germany
Email Address: mian.dai@tu-darmstadt.de
Xiaoqing Li, Stephan Schönecker
Department of Materials Science and Engineering, KTH - Royal Institute of Technology, SE-10044, Stockholm, Sweden
Liuliu Han
Max Planck Institute for Sustainable Materials, Max-Planck-Str. 1, Düsseldorf, Germany

Δ-test between EMTO and VASP

The Δ-test, as introduced by Lejaeghere et al. [1], provides a standardized approach for DFT calculations. It evaluates the root mean square deviation of the equation of state (EOS) over a predefined volume range, enabling the comparison of computational precision between methods.
To assess the precision of our EMTO calculations, we performed a Δ-test comparing EMTO with the Vienna Ab initio Simulation Package (VASP)[2, 3] and the all-electron WIEN2k method[4] as summarized in Table 1. For BCC structures, the Δ-value between EMTO and VASP is 1.113, and for FCC structures, it is 1.331, yielding an average Δ-value of 1.222. Against WIEN2k, the Δ-value is 1.168. These low Δ-values underscore the consistency of EMTO with well-established methods, validating its reliability in predicting structural and energetic properties.

Table 1: Averaged volumes and Δ values of 42 single elements in BCC and FCC structures for comparison between the EMTO and VASP methods.

	BCC	FCC	Total
V-emto [$\AA^3$]	19.487	19.697	19.592
V-vasp [$\AA^3$]	19.258	19.428	19.343
Δ	1.113	1.331	1.222
V-emto [$\AA^3$]			14.083
V-wien2k [$\AA^3$]			14.086[1]
Δ			1.168

Validation of Phase Stability and Lattice Constant

Table 2 provides a comprehensive comparison between the HTP EMTO-CPA calculations and the experimentally reported data for various HEA systems. These systems include compositions with elements such as Al, Cr, Co, Cu, Fe, Mn, Mo, Ni, Ti, V, and Zn. The table details the predicted phases (BCC, FCC, or FCC+BCC) and calculated lattice parameters, along with the corresponding experimental phases and lattice constants, where available.
The comparison reveals a high degree of consistency between the calculated EMTO-CPA results and experimental observations, particularly in terms of phase stability and lattice parameters. For example, the predicted phases for most HEA compositions align well with the experimentally determined phases, and the calculated lattice constants closely match the reported values. This agreement underscores the accuracy and reliability of the EMTO-CPA method for modeling the structural properties of HEAs.

Validation of Bulk Modulus

To validate the precision of our HTP EMTO-CPA calculations, we compare the calculated bulk moduli (B_0) of various HEAs with values reported in the literature using the coherent potential approximation (CPA) and supercell methods. The results are summarized in Table 3, demonstrating the reliability of our computational approach.
The calculated bulk modulus (B_0) for each HEA system is in excellent agreement with the previously reported values of CPA (B_0^*) and supercell (B_0^{**}). For example, the bulk modulus of CrFeCoNi in the FCC phase is 195.5 GPa, closely matching the CPA-reported value of 207.0 GPa and the supercell value of 208 GPa. Similarly, other HEA systems, such as MnCrCoNi and CrNiMoW, exhibit comparable consistency between our calculated values and those reported in the literature.
This consistency across different HEA systems confirms the robustness and reliability of the EMTO-CPA method to determine the elastic properties of HEAs. In line with established computational approaches, including CPA and supercell methods, our high-throughput calculations provide a reliable framework for exploring the elastic properties of complex alloys, supporting their use in further predictive studies and material design efforts.

Validation of Curie Temperature

Table 4 presents a comparison of high-throughput Curie temperatures computed with EMTO-CPA (T_c) with previously reported computational (T_c^*) and experimental (T_c^{**}) values for various HEA systems. The results indicate strong agreement, validating the reliability of the EMTO-CPA method for predicting magnetic properties.
For example, AlFeCoNi in the BCC phase has a calculated T_c of 779 K, closely matching the reported computational value of 763 K. Similarly, FeCoCuNi in the FCC phase shows a computed T_c of 830 K, consistent with experimental data of 826 K and a computational value of 796 K. Cases like CrFeCoNi also demonstrate close alignment, with the calculated T_c of 127 K falling within the experimental range of 120–130 K.
In general, the discrepancies are minimal and within acceptable margins, highlighting the robustness of the EMTO-CPA method in accurately predicting the T_c values for the HEA

Table 2: Comparison of HTP EMTO-CPA results with reported experimental values [5, 6, 7, 8, 9, 10, 11, 12, 13, 14, 15, 16, 17, 18, 19, 20, 21, 22, 23, 24, 25, 26, 27, 28, 29, 30] (*) for HEA systems.

System	Phase	Lattice parameter (Å)	Phase *	Lattice parameter * (Å)
AlCrFeNi	BCC	2.882	BCC	-
TiAlCuNi	BCC	-	FCC	-
CrFeCoCu	FCC	3.559	FCC	-
FeCoNiPd	FCC	3.700	FCC	-
VFeCoNi	FCC	3.575	FCC	-
CoNiRuRh	FCC	3.726	FCC	-
MnCrFeNi	FCC	3.535	FCC	-
TiAlFeCoNi	BCC	2.939	BCC	-
TiAlCrMoW	BCC	3.144	BCC	-
TiAlFeCuNi	BCC	-	FCC	-
MnAlCuNiPt	FCC	3.798	FCC	-
ZnCrCoCuNi	FCC	3.612	FCC	-
TiCrFeCoNi	BCC	-	FCC	-
MnFeCoCuNi	BCC	-	FCC	-
FeCoCuNiMo	FCC	3.691	FCC	-
FeCoCuNiPd	FCC	-	BCC	
FeCoCuNiPt	FCC	3.721	FCC	-
TiFeCoCuNi	FCC	3.650	FCC	-
CoCuNiPdPt	FCC	3.790	FCC	-
FeCoPdIrPt	FCC	3.848	FCC	-
FeCoNiPdPt	FCC	3.785	FCC	-
CrFeCuNiMo	BCC	-	FCC	-
CrFeCoNi	FCC	3.552	FCC	3.568
MnCrCoNi	FCC	3.527	FCC	3.601
MnCoCuNi	FCC	3.618	FCC	3.586
AlCoCuNi	FCC	3.620	FCC+BCC	3.603
MnFeCoNi	FCC	3.550	FCC	3.600
FeCoCuNi	FCC	3.576	FCC	3.586
ZrTiNbCr	BCC	3.287	BCC	3.365
TiAlCrMo	BCC	3.098	BCC	3.101
NbCrCoMo	BCC	3.073	BCC	3.147
VCrFeMo	BCC	2.979	BCC	2.999
AlFeCoNi	BCC	2.868	BCC	2.867
AlCrCoCuNi	BCC	3.614	FCC+BCC	3.588
AlFeCoCuNi	BCC	3.619	FCC+BCC	3.615
CrFeCoCuNi	FCC	3.573	FCC	3.578
CrFeCoNiPd	FCC	3.672	FCC	3.648
AlCrFeCoNi	BCC	2.863	FCC+BCC	2.866
MnCrFeCoNi	BCC	2.839	FCC+BCC	2.858

Table 3: Comparison of HTP EMTO-CPA bulk moduli with reported computational CPA (*) [31, 32, 33, 34, 35, 36, 37, 38, 39] and supercell (**) for HEAs.

System	Phase	B_0 (GPa)	B_0^* (GPa)	B_0^{**} (GPa)
CrFeCoNi	FCC	195.5	207.0	208
MnCrCoNi	FCC	176.2	190.8	
MnFeCoNi	BCC	147.4	149.2	
MnCrMoW	BCC	217.9	202.4	
CrNiMoW	BCC	224.5	245.0	
FeCoCuNi	FCC	181.7	175.0	
TiVCrMo	BCC	181.2	195.5	
VFeCoCuNi	FCC	176.1	173.0	
CrFeCoCuNi	FCC	175.8	179.0	
MnFeCoCuNi	BCC	142.1	144.0	

systems.

Machine Learning Models

Table 5 summarizes the performance of various machine learning models in predicting the target properties of high-entropy alloys (HEA), namely the bulk modulus (B_0), magnetic moment per unit cell (M_s) and Curie temperature (T_c). The table provides R^2 scores for both training and testing sets, highlighting the generalization of each model and the predictive accuracy.
Among the models evaluated, LightGBMLarge achieved the highest performance in all target properties, with R^2 test scores of 0.917 for B_0, 0.996 for M_m, and 0.939 for T_c. These results underscore its strong predictive power and robustness. Similarly, RandomForestMSE and ExtraTreesMSE delivered high accuracy, with R^2 test scores exceeding 0.9 for most properties. Competitive performance was also observed for CatBoost and XGBoost, with test scores R^2 of 0.905 and 0.908 for B_0, respectively, and comparable values for predictions M_s and T_c.
In contrast, simpler models, such as KNeighborsUnif and KNeighborsDist, showed significantly lower predictive capabilities, with R^2 test scores below 0.9 for B_0 and negative scores for M_m and T_c, indicating their limitations in capturing the complexity of the underlying relationships in the data set.
The weighted assembly approach demonstrated consistently high performance in all properties, with test scores of R^2 of 0.921 for B_0, 0.986 for M_m, and 0.943 for T_c. This result highlights the effectiveness of combining multiple models to improve prediction accuracy.
In summary, the analysis in Table 5 shows that advanced ensemble and tree-based models, such as LightGBMLarge, ExtraTreesMSE and WeightedEnsemble, are particularly effective in predicting the properties of HEA. In contrast, simpler models may lack the complexity necessary to accurately capture these intricate relationships.

Table 4: Comparison of HTP EMTO-CPA Curie temperatures with reported computational (*) [40] and experimental values (**) [41, 23, 42, 19, 43, 44, 45, 46] for HEAs.

System	Phase	Tc (K)	Tc * (K)	Tc ** (K)
AlFeCoNi	BCC	779	763	
CrFeCoNi	FCC	127	155	120-130
AlCoCuNi	BCC	250	245	
MnFeCoNi	FCC	171	166	
FeCoCuNi	FCC	830	796	826
TiAlFeCuNi	FCC	209	213	
TiAlFeCuNi	BCC	295	286	
AlCrFeCoNi	FCC	127	136	
AlCrFeCoNi	BCC	342	334	
AlCrFeCuNi	FCC	124	124	
AlCrFeCuNi	BCC	165	159	
AlCrCoCuNi	FCC	20	25	
AlCrCoCuNi	BCC	71	70	
AlFeCoCuNi	FCC	503	493	
TiVCrFeMo	BCC	144	97	
TiCrFeCoNi	FCC	83	95	
TiCrFeCoNi	BCC	346	339	
VFeCoCuNi	FCC	331	246	
MnCrFeCuNi	FCC	32	60	
MnCrFeCuNi	BCC	197	191	
MnCrFeCoNi	FCC	88	27	20
CrFeCoCuNi	FCC	244	251	172
CrFeCoNiPd	FCC	425	440	440
CrFeCoNiMo	FCC	122	102	
MnFeCoCuNi	BCC	556	540	400
MnFeCoNiMo	FCC	57	68	
FeCoCuNiMo	FCC	544	328	657
FeCoCuNiAg	FCC	816	805	
FeCoCuNiPt	FCC	858	837	864

Table 5: Performance of various base models on target properties.

Model	B_0		M_s		T_c	
	R^2 train	R^2 test	R^2 train	R^2 test	R^2 train	R^2 test
KNeighborsUnif	0.884	0.815	0.249	-0.090	0.249	-0.089
NeuralNetTorch	0.902	0.888	0.990	0.984	0.911	0.901
NeuralNetFastAI	0.923	0.908	0.989	0.985	0.938	0.925
KNeighborsDist	0.959	0.840	0.381	-0.502	0.402	-0.468
CatBoost	0.967	0.914	0.995	0.983	0.981	0.935
XGBoost	0.970	0.908	0.995	0.980	0.984	0.927
WeightedEnsemble	0.974	0.921	0.995	0.988	0.987	0.943
LightGBMXT	0.975	0.916	0.997	0.986	0.984	0.939
LightGBM	0.980	0.916	0.998	0.986	0.982	0.936
RandomForestMSE	0.982	0.899	0.997	0.983	0.986	0.924
ExtraTreesMSE	0.983	0.908	0.997	0.984	0.986	0.926
LightGBMLarge	0.985	0.917	0.999	0.988	0.993	0.939

Table 6: Feature descriptions for material properties

**Feature**	**Property**	**Unit**
AtomicRadius	Atomic radius	$\AA^3$
AtomicVolume	Volume of an atom of each element	$\AA^3$ / atom
AtomicWeight	Atomic weight	-
Column	Column on periodic table	-
CovalentRadius	Covalent radius of each element	pm
Electronegativity	Pauling electronegativity	-
GSbandgap	DFT bandgap energy of $T = 0K$ ground state	eV
GSmagmom	DFT magnetic moment of $T = 0K$ ground state	-
GSvolume_pa	DFT volume per atom of $T = 0K$ ground state	$\AA^3$ / atom
MeltingT	Melting temperature of element	K
MendeleevNumber	Mendeleev Number	-
NdUnfilled	Number of unfilled d valence orbitals	-
NdValence	Number of filled d valence orbitals	-
NfUnfilled	Number of unfilled f valence orbitals	-
NfValence	Number of filled f valence orbitals	-
NpUnfilled	Number of unfilled p valence orbitals	-
NpValence	Number of filled p valence orbitals	-
NsUnfilled	Number of unfilled s valence orbitals	-
NsValence	Number of filled p valence orbitals	-
Row	Row on periodic table	-

SpaceGroupNumber	Space group of $T = 0K$ ground state structure	-
min_{feature}	Minimum value of the properties	-
max_{feature}	Maximum value of the properties	-
maxdiff_{feature}	Maximum difference in the properties	-
mean_{feature}	Average value of the properties	-
dev_{feature}	Standard deviation of the properties	-
most_{feature}	Most frequently occurring in the properties	-

References

[1] K. Lejaeghere, G. Bihlmayer, T. Björkman, P. Blaha, S. Blügel, V. Blum, D. Caliste, I. E. Castelli, S. J. Clark, A. Dal Corso, p. u. family=Gironcoli, given=Stefano, T. Deutsch, J. K. Dewhurst, I. Di Marco, C. Draxl, M. Duł ak, O. Eriksson, J. A. Flores-Livas, K. F. Garrity, L. Genovese, P. Giannozzi, M. Giantomassi, S. Goedecker, X. Gonze, O. Grå näs, E. K. U. Gross, A. Gulans, F. c. Gygi, D. R. Hamann, P. J. Hasnip, N. A. W. Holzwarth, D. Iu¸san, D. B. Jochym, F. c. Jollet, D. Jones, G. Kresse, K. Koepernik, E. Kü¸cükbenli, Y. O. Kvashnin, I. L. M. Locht, S. Lubeck, M. Marsman, N. Marzari, U. Nitzsche, L. Nordström, T. Ozaki, L. Paulatto, C. J. Pickard, W. Poelmans, M. I. J. Probert, K. Refson, M. Richter, G.-M. Rignanese, S. Saha, M. Scheffler, M. Schlipf, K. Schwarz, S. Sharma, F. Tavazza, P. Thunström, A. Tkatchenko, M. Torrent, D. Vanderbilt, p. u. family=Setten, given=Michiel J., V. Van Speybroeck, J. M. Wills, J. R. Yates, G.-X. Zhang, S. Cottenier, *Science* **2016**, *351*, 6280 aad3000.

[2] P. E. Blöchl, *Physical Review B* **1994**, *50*, 24 17953.

[3] G. Kresse, D. Joubert, *Physical Review B* **1999**, *59*, 3 1758.

[4] G. K. H. Madsen, P. Blaha, K. Schwarz, E. Sjöstedt, L. Nordström, *Physical Review B* **2001**, *64*, 19 195134.

[5] T. Zuo, M. C. Gao, L. Ouyang, X. Yang, Y. Cheng, R. Feng, S. Chen, P. K. Liaw, J. A. Hawk, Y. Zhang, *Acta Materialia* **2017**, *130* 10.

[6] T. T. Zuo, R. B. Li, X. J. Ren, Y. Zhang, *Journal of Magnetism and Magnetic Materials* **2014**, *371* 60.

[7] Y. X. Zhuang, W. J. Liu, Z. Y. Chen, H. D. Xue, J. C. He, *Materials Science and Engineering: A* **2012**, *556* 395.

[8] Y. Zhang, Y. J. Zhou, J. P. Lin, G. L. Chen, P. K. Liaw, *Advanced Engineering Materials* **2008**, *10*, 6 534.

[9] M. Zhang, X. Zhou, J. Li, *Journal of Materials Engineering and Performance* **2017**, *26*.

[10] K. B. Zhang, Z. Y. Fu, J. Y. Zhang, W. M. Wang, H. Wang, Y. C. Wang, Q. J. Zhang, J. Shi, *Materials Science and Engineering: A* **2009**, *508*, 1 214.

[11] J.-W. Yeh, S.-Y. Chang, Y.-D. Hong, S.-K. Chen, S.-J. Lin, *Materials Chemistry and Physics* **2007**, *103*, 1 41.

[12] J.-W. Yeh, S.-K. Chen, S.-J. Lin, J.-Y. Gan, T.-S. Chin, T.-T. Shun, C.-H. Tsau, S.-Y. Chang, *Advanced Engineering Materials* **2004**, *6*, 5 299.

[13] Y. D. Wu, Y. H. Cai, T. Wang, J. J. Si, J. Zhu, Y. D. Wang, X. D. Hui, *Materials Letters* **2014**, *130* 277.

[14] N. D. Stepanov, D. G. Shaysultanov, G. A. Salishchev, M. A. Tikhonovsky, *Materials Letters* **2015**, *142* 153.

[15] O. N. Senkov, S. V. Senkova, C. Woodward, D. B. Miracle, *Acta Materialia* **2013**, *61*, 5 1545.

[16] G. A. Salishchev, M. A. Tikhonovsky, D. G. Shaysultanov, N. D. Stepanov, A. V. Kuznetsov, I. V. Kolodiy, A. S. Tortika, O. N. Senkov, *Journal of Alloys and Compounds* **2014**, *591* 11.

[17] F. Otto, Y. Yang, H. Bei, E. P. George, *Acta Materialia* **2013**, *61*, 7 2628.

[18] A. Marshal, K. G. Pradeep, D. Music, S. Zaefferer, P. S. De, J. M. Schneider, *Journal of Alloys and Compounds* **2017**, *691* 683.

[19] M. S. Lucas, L. Mauger, J. A. Muñoz, Y. Xiao, A. O. Sheets, S. L. Semiatin, J. Horwath, Z. Turgut, *Journal of Applied Physics* **2011**, *109* 07E307.

[20] L. Liu, J. B. Zhu, C. Zhang, J. C. Li, Q. Jiang, *Materials Science and Engineering: A* **2012**, *548* 64.

[21] C. Li, J. C. Li, M. Zhao, Q. Jiang, *Journal of Alloys and Compounds* **2009**, *475*, 1 752.

[22] G. Laplanche, P. Gadaud, O. Horst, F. Otto, G. Eggeler, E. P. George, *Journal of Alloys and Compounds* **2015**, *623* 348.

[23] M. Kurniawan, A. Perrin, P. Xu, V. Keylin, M. McHenry, *IEEE Magnetics Letters* **2016**, *7* 1.

[24] S. Jiang, D. sun, Y. Zhang, S. Wang, C. Zhao, *Journal of Materials Science* **2017**, *52*, 6 3199.

[25] J. Guo, X. Huang, W. Huang, *Journal of Materials Engineering and Performance* **2017**, *26*, 7 3071.

[26] H.-P. Chou, Y.-S. Chang, S.-K. Chen, J.-W. Yeh, *Materials Science and Engineering: B* **2009**, *163*, 3 184.

[27] S. Y. Chen, X. Yang, K. A. Dahmen, P. K. Liaw, Y. Zhang, *Entropy* **2014**, *16*, 2 870.

[28] H. Chen, A. Kauffmann, S. Laube, I.-C. Choi, R. Schwaiger, Y. Huang, K. Lichtenberg, F. Müller, B. Gorr, H.-J. Christ, M. Heilmaier, *Metallurgical and Materials Transactions A* **2017**.

[29] B. Cantor, I. T. H. Chang, P. Knight, A. J. B. Vincent, *Materials Science and Engineering: A* **2004**, *375–377* 213.

[30] N. Adomako, J. Kim, Y. Hyun, *Journal of Thermal Analysis and Calorimetry* **2018**, *133*.

[31] F. Tian, L. Varga, N. Chen, J. Shen, L. Vitos, *Journal of Alloys and Compounds* **2014**, *599* 19.

[32] X. Li, F. Tian, S. Schönecker, J. Zhao, L. Vitos, *Scientific Reports* **2015**, *5*, 1 12334.

[33] L.-Y. Tian, G. Wang, J. S. Harris, D. L. Irving, J. Zhao, L. Vitos, *Materials & Design* **2017**, *114* 243.

[34] F. Tian, L. Varga, J. Shen, L. Vitos, *Computational Materials Science* **2016**, *111* 350.

[35] H. Ge, H. Song, J. Shen, F. Tian, *Materials Chemistry and Physics* **2017**, *210*.

[36] H. Ge, F. Tian, Y. Wang, *Computational Materials Science* **2017**, *128* 185.

[37] F. Tian, L. Varga, L. Vitos, *Intermetallics* **2017**, *83* 9.

[38] F. Tian, L. Delczeg, N. Chen, L. K. Varga, J. Shen, L. Vitos, *Physical Review B* **2013**, *88*, 8 085128.

[39] S. Huang, A. Vida, A. Heczel, E. Holmstrom, L. Vitos, *JOM* **2017**, *69*, 11 2107.

[40] S. Huang, E. Holmström, O. Eriksson, L. Vitos, *Intermetallics* **2018**, *95* 80.

[41] D. Ma, B. Grabowski, F. Körmann, J. Neugebauer, D. Raabe, *Acta Materialia* **2015**, *100* 90.

[42] Y.-F. Kao, S.-K. Chen, T.-J. Chen, P.-C. Chu, J.-W. Yeh, S.-J. Lin, *Journal of Alloys and Compounds* **2011**, *509*, 5 1607.

[43] X. F. Wang, Y. Zhang, Y. Qiao, G. L. Chen, *Intermetallics* **2007**, *15*, 3 357.

[44] C. Niu, A. J. Zaddach, A. A. Oni, X. Sang, J. W. Hurt, III, J. M. LeBeau, C. C. Koch, D. L. Irving, *Applied Physics Letters* **2015**, *106*, 16 161906.

[45] M. S. Lucas, D. Belyea, C. Bauer, N. Bryant, E. Michel, Z. Turgut, S. O. Leontsev, J. Horwath, S. L. Semiatin, M. E. McHenry, C. W. Miller, *Journal of Applied Physics* **2013**, *113*, 17 17A923.

[46] F. Körmann, D. Ma, D. Belyea, M. Lucas, C. Miller, B. Grabowski, M. Sluiter, *Applied Physics Letters* **2015**, *107*.
